# Lessons Learned From a Healthful Vending Pilot Program in Delaware State Agency Buildings, 2011–2012

**DOI:** 10.5888/pcd11.140188

**Published:** 2014-08-21

**Authors:** Laura Lessard, Mollie Poland, Mary Trotter

**Affiliations:** Author Affiliations: Laura Lessard, Arcadia University, Glenside, Pennsylvania, and Nemours Health and Prevention Services, Wilmington, Delaware; Mary Trotter, Nemours Health and Prevention Services, Wilmington, Delaware.

## Abstract

**Introduction:**

Changes in food availability in worksites can result in changes in eating behavior and weight status. Nemours Health and Prevention Services, in conjunction with partners in Delaware, conducted a 6-month pilot program to assess the feasibility and impact of requiring that 75% of the items in vending machines in 3 state agency buildings have healthful items.

**Methods:**

We collected process evaluation data from October 2011 through April 2012 by taking weekly photographs of all machines to record the number of healthful items available. Outcomes were measured through sales reports designed to enumerate changes in number and type of items sold and overall profit from each building.

**Results:**

We found challenges in fully implementing the 75% goal. In one of the 3 buildings, all machines were compliant within 7 weeks; in another, full compliance did not occur until week 19. Despite these challenges, the number of items sold in each machine was comparable to numbers from the previous year. Total profits from each building varied across the 3 sites and during the pilot. One building had a 51% increase in profits in January 2012 compared with profits averaged for January 2011 and January 2010. In contrast, monthly profit at another building fluctuated from an increase of 6% to a loss of 30%.

**Conclusion:**

Overall, our results suggest that collaborative efforts can result in a feasible intervention with little negative influence on profits.

## Introduction

In Delaware and throughout the United States, rates of overweight and obesity are high and are continuing to rise. According to the Centers for Disease Control and Prevention, 63.4% of Delaware’s adult population in 2010 was overweight (body mass index [BMI] ≥25.0) and 28.0% was obese (BMI ≥30.0) (1). Overweight and obesity are significant risk factors for numerous life-threatening chronic conditions, including diabetes, cardiovascular disease, and several types of cancer (2). Poor nutrition (ie, consuming excessive amounts of foods and beverages high in calories, sugar, and fat) and insufficient amounts of physical activity contribute to overweight and obesity (3).

Worksite-based interventions designed to improve employee health are a possible method to address obesity (4,5). People consume a significant amount of their daily total calories while at work; one study suggested that adults consume 20% of calories from sugar-sweetened beverages at work (6). The nutritional quality of foods and beverages sold in vending machines historically has been poor. Items commonly sold in conventional vending machines are generally high in calories, sugar, and saturated fat, thus contributing to poor eating habits among many adults (7,8).

Recognizing vending machines as an important venue for promoting nutritional choices that are more healthful than those typically offered, Nemours Health and Prevention Services (NHPS), the health promotion and disease prevention arm of Nemours pediatric health system, partnered with the Delaware Division of Public Health and the state licensing agency responsible for the oversight and implementation of the Delaware Division of the Visually Impaired Business Enterprise Program. This partnership developed and implemented a 6-month healthful food and beverage vending program at 3 pilot sites in Delaware. 

The goal of the pilot program was to increase the availability of healthful vended items, maximize consumer choice for these items, and educate consumers on nutrition. The objective of this evaluation was to answer the following questions:

1. Has the program improved the nutritional quality of the food and beverage items available to employees and visitors at pilot sites?
2. To what extent have employees and visitors purchased more healthful items?
3. Have changes in the nutritional quality of vended foods and beverages affected the revenue from machines at pilot sites?

## Methods

### Development of the pilot program

The 3 pilot sites were chosen to represent the diversity of Delaware: one building in a northern urban location, one building in a central location on the legislative mall in the state’s capitol city, and one building in a southern rural location. The northern building housed one food machine and 4 beverage machines, all of which were included in the program; the central building had one food and 2 beverage machines, but one of the beverage machines was not included in the program; and the southern building had 2 food and 2 beverage machines, but only the food machines were included. The Division of Public Health and the Business Enterprise Program are part of Delaware’s Department of Health and Social Services, and the pilot sites are Department of Health and Social Services office buildings.

The pilot program began with an assessment of employee interest and preferences for food and beverage items. After surveying employees at the 3 sites and conducting on-site taste tests of potential new items, the selection of foods and beverages was adjusted in favor of more healthful alternatives.

The pilot program ran for 28 weeks, from October 26, 2011 through April 24, 2012. The program required that at least 75% of the items in all vending machines at the pilot sites meet NHPS’ “Go” or “Slow” food guidelines and that no more than 25% would be “Whoa” items. The 75% goal was set by the director of the Division of Public Health, and contracts were put into place between parties that reflected this goal and the overall goals of the project. The Go, Slow, and Whoa nutrition guidelines (9) were developed by NHPS in 2010 on the basis of current science (eg, Dietary Guidelines for Americans, recommendations by the Institute of Medicine for schools). Go or Slow items must contain no more than 200 total calories, 35% of calories from fat, 10% of calories from saturated fat, and 200 mg of sodium. Nuts and seeds are exempt from these requirements because of their fiber, vitamin E, and superior fat content; however, these items must meet the criteria for sodium and calories. Additionally, Go and Slow foods must contain no trans fats, and candy is not allowed. All foods that do not meet these criteria are classified as Whoa foods. Go beverages consist of water without added flavoring or additives; Slow beverages consist of 100% fruit juices or contain no more than 10 total calories per 8-oz serving (eg, diet sodas and teas, flavored water).

Selection of more healthful items was also based on a subset of the Canteen Vending Services’ Balanced Choice Options that meet NHPS guidelines (10). New items included baked chips, 100-calorie packs of popular cookies and crackers, trail mix, energy bars, diet soda and diet tea, and flavored water. Similar options were offered at each site, although some adjustments were made in response to preferences expressed via taste tests or surveys. To encourage employees to purchase the more healthful items, these items were marked with a special symbol and promoted with signage on or around the machines. Additional marketing of the pilot program and the new items and nutrition information was shared in a series of 5 e-mail messages to staff at each site and in a newsletter for all state employees.

Because of concerns over revenue loss, a monetary safeguard agreement was established between the Business Enterprise Program and the Division of Public Health. The Division of Public Health agreed to reimburse vendors for any monthly losses incurred during the pilot program; a loss was defined as an amount less than the average gross revenue for the same month in the previous 2 years.

### Evaluation of the pilot program

One key assumption of the evaluation was that 2 markets of employees were potentially affected by this program: those who had been purchasing the less healthful items from the vending machines and those who had not been purchasing any items from the machines. The program was designed to change the buying behavior (via changes in access to more healthful items) of the former market and encourage the latter market to use the machines. 

The evaluation used existing data (eg, monthly sales reports) wherever possible to reduce burden on participants. Additional data were collected via photographic documentation of the vending machines. These data were supplemented with online surveys and interviews of stakeholders, including but not limited to drivers, operators, and employees at the pilot sites; this evaluation does not include the findings of the interviews or the surveys.

To objectively document the contents of the vending machines during the study period, each machine was photographed weekly by a trained building employee. The photographs were then sent to NHPS and converted into planograms, diagrams that provide details on placement of products in retail environments. The planograms were used to determine whether machines complied with the 75% goal, assess the number and type of sold-out items, and confirm pricing. If an item was out of stock or the machine did not meet the 75% goal, the study team contacted the Business Enterprise Program to remedy the problem. Data were recorded in a spreadsheet and tracked during the 28-week study period.

Monthly sales reports included information about each item sold in each machine and the amount of revenue generated. We also had access to data on items sold during the same 28-week period in the previous year and access to data on monthly profits made by each building during the study period and during the previous 2 years.

### Analysis

We calculated the proportion of Go and Slow items in each machine to determine compliance on a weekly basis at each site. We also analyzed the number and proportion of Go, Slow, and Whoa items in each machine according to the following 5 periods: period 3, November 23 through December 27; period 4, December 28 through January 24; period 5, January 25 through February 21; period 6, February 22 through March 27; and Period 7, March 28 through April 24. We compared the total number of items sold in each period with the number of items sold during the same period in the previous year. Although the beverage machines in the southern location were exempt from the contract with the Business Enterprise Program, the vendors were permitted to stock the machines with the more healthful options, and we included these machines in our analysis.

We calculated the monthly profit at each site for 6 months beginning with November 2011. We compared these monthly profits with the profits made in the same months in the 2 years before the pilot; for example, November 2011 was compared with November 2010 and November 2009. Because of the limited availability of data, we could not compare the profits of the pilot sites with the profits of sites not participating in the pilot program; these data could have explained any changes in sales that were not caused by the pilot (eg, normal fluctuations in sales of vending items, the economy). 

## Results

### Has the program improved the nutritional quality of the foods and beverages available to employees and visitors at the pilot sites?

**Figure 1:**
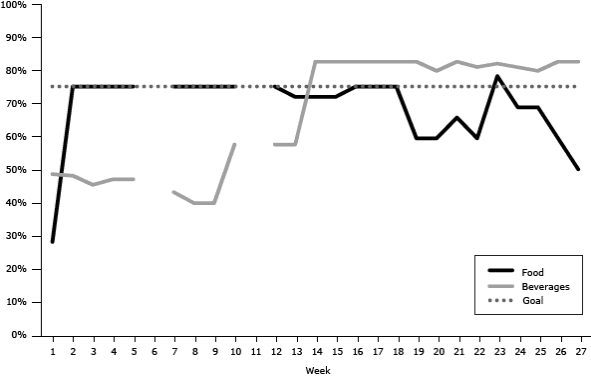
Percentage of Go and Slow foods and beverages available in vending machines by week, northern pilot program site, Delaware state agency buildings, 2011–2012. The values for beverages are averages for the 4 beverage machines. Breaks in data indicate weeks for which data were unavailable. **Table:** 

In several buildings, we faced challenges in bringing the machines into compliance with the 75% goal and in continuing to achieve this goal throughout the pilot. At the beginning of the pilot program at the northern site, only 28% of the food items and 49% of the beverage items were Go or Slow items. By the third week, 75% of the items in the food machine were more healthful items, but the beverage machines were not fully compliant until after week 13 (Figure 1).

Week Number	Food Items, %	Beverages, %
1	28	49
2	75	48
3	75	45
4	75	47
5	75	47
6	—	—
7	75	43
8	75	39
9	75	40
10	75	58
11	—	—
12	75	58
13	72	58
14	72	82
15	72	82
16	75	82
17	75	82
18	75	82
19	59	82
20	59	80
21	66	82
22	59	81
23	78	82
24	69	81
25	69	80
26	59	82
27	50	82

At the central location, at the beginning of the pilot program, only 25% of the food items and 12% of the beverage items were Go or Slow items. By the third week, 75% of the items in the food machine were more healthful items, but the beverage machine was not fully compliant until week 19. 

**Figure 2:**
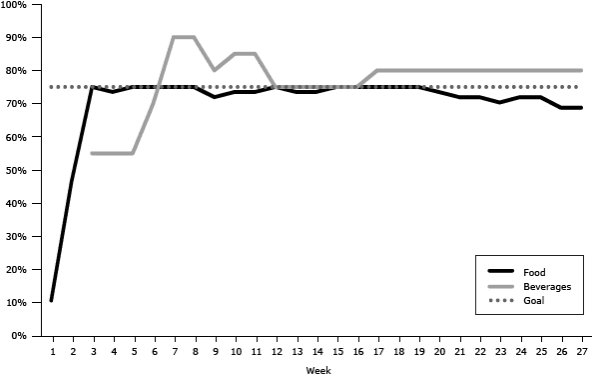
Percentage of Go and Slow foods and beverages available in vending machines by week, southern pilot program site, Delaware state agency buildings, 2011–2012. The values for beverages are averages for all vending machines. Breaks in data indicate weeks for which data were unavailable. **Table:** 

At the beginning of the pilot program, only 11% of the food items and 50% of the beverage items were Go or Slow items (Figure 2) at the southern site. By the third week, 75% of items in the food machines were more healthful items, but the beverage machines were not fully compliant until after week 6.

Week Number	Food Items, %	Beverages, %
1	11	50
2	46	—
3	75	55
4	74	55
5	75	55
6	75	70
7	75	90
8	75	90
9	72	80
10	74	85
11	74	85
12	75	75
13	74	75
14	74	75
15	75	75
16	75	75
17	75	80
18	75	80
19	75	80
20	74	80
21	72	80
22	72	80
23	70	80
24	72	80
25	72	80
26	69	80
27	69	80

### To what extent have employees and visitors purchased more healthful items?

In general, we found increases in the total number of items purchased during the pilot program compared with the previous year. For example, at the northern site, the number of items purchased in periods 5 through 7 (n = 434) (Table 1) was higher than the number of items purchased during the same periods in the previous year (n = 305). We found a 16% increase in purchases from 2011 to 2012 for period 5, a 68% increase for period 6, and a 43% increase for period 7.

**Table 1:** No. (%)^a^ of “Go,” “Slow,” and “Whoa” Food and Beverage^b^ Items Sold in Vending Machines in Pilot Program, Delaware State Agency Buildings, 2011–2012

Item type^b^	Period 3 (11/23–12/27)	Period 4 (12/28–1/24)	Period 5 (1/25–2/21)	Period 6 (2/22–3/27)	Period 7 (3/28–4/24)
**Food Items**					
**Northern site**					
Slow	77 (56)	51 (65)	72 (59)	124 (71)	99 (72)
Whoa	60 (44)	28 (35)	50 (41)	51 (29)	38 (28)
Total, n	137	79	122	175	137
**Central site**					
Slow	55 (49)	52 (44)	64 (53)	97 (53)	83 (52)
Whoa	57 (51)	65 (56)	57 (47)	86 (47)	78 (48)
Total, n	112	117	121	183	161
**Southern site**					
Slow	181 (54)	91 (47)	133 (48)	217 (49)	127 (47)
Whoa	157 (46)	104 (53)	147 (52)	224 (51)	141 (53)
Total, n	338	195	280	441	268
**Beverage Items**					
**Northern**					
Go	35 (11)	26 (10)	16 (7)	22 (6)	22 (7)
Slow	131 (42)	85 (34)	121 (51)	189 (55)	159 (50)
Whoa	149 (47)	139 (56)	98 (42)	130 (38)	135 (43)
Total, n	315	250	235	341	316
**Central**					
Go	3 (3)	3 (3)	2 (2)	9 (6)	1 (1)
Slow	58 (60)	62 (72)	75 (69)	80 (56)	42 (51)
Whoa	35 (36)	21 (24)	31 (29)	55 (38)	40 (48)
Total, n	96	86	108	144	83

a Percentages may not total 100% because of rounding.

b Categories of “Go,” “Slow,” and “Whoa” items established by Nemours Health and Prevention Services (9). Slow food items contain no more than 200 total calories, 35% of calories from fat, 10% of calories from saturated fat, and 200 mg of sodium. Nuts and seeds are exempt because of their fiber, vitamin E, and superior fat content; however, these items meet criteria for sodium and calories. Go beverages consist of water without added flavoring or additives. Slow beverages consist of 100% fruit juice or contain no more than 10 total calories per 8-oz serving (eg, diet sodas and teas, flavored water). All items not classified as Slow or Go are classified as Whoa.

At the northern site, although the number of items purchased varied during the pilot program, the proportion of purchases of Slow foods increased from 56% in period 3 to 72% in period 7 (Table 1). At the central and southern sites, purchases were fairly evenly split between Slow foods and Whoa foods. In one machine in the southern location, the best-seller (according to number of items purchased) for 3 of the 5 periods was a Slow food.

At the northern site, the proportion of purchases of Go and Slow beverages increased during the pilot period (Table 1). At the central site, we found initial increases in the proportion of more healthful beverages purchased, but by the end of the pilot program, that proportion decreased to 52% (from 63% during the first month).

### Have changes in the nutritional quality of vended foods and beverages affected the revenue from machines in pilot sites?

Among the 3 sites, gains (compared with the guarantees) in monthly profits ranged from 4% ($27.68) at the southern site in March 2012 to 51% ($356.58) at the same site in January 2012. Losses ranged from −4% (−$16.74) at the central site in December 2011 to −36% (−$170.99) at the same site in January 2012 (Table 2). Overall, profits at the southern site were greater than the guarantees for 5 of 6 months of the pilot.

**Table 2:** Net Gain or Loss in Total Profits for Participating Vending Machines Compared With Guarantees for Each Pilot Site, Delaware State Agency Buildings, 2011–2012

Site	Net Gain or Loss, $ (%)					
	Nov 2011	Dec 2011	Jan 2012	Feb 2012	Mar 2012	Apr 2012
Northern	−99.63 (−17)	−35.88 (−6)	−181.73 (−30)	−159.03 (−27)	38.62 (6)	−33.13 (−6)
Central	−93.39 (−20)	−16.74 (−4)	−170.99 (−36)	−61.09 (−13)	−95.34 (−20)	−134.74 (−29)
Southern	88.23 (13)	282.33 (40)	356.58 (51)	−60.67 (−9)	27.68 (4)	213.08 (30)

## Discussion

Our experience of piloting healthful vending in Delaware resulted in numerous successes, lessons learned, and areas of opportunity. The program was conducted as a collaboration of public health interests (the Department of Health and Social Services and NHPS) and entrepreneurial business interests (the vending machine operators). To effectively execute the pilot program, it was critical to understand that each stakeholder was uniquely motivated. Likewise, each stakeholder group needed to have their concerns heard, validated, and woven into the negotiations on pilot parameters. Addressing these unique motivations and divergent perspectives was challenging but essential throughout the process — even getting the pilot off the ground. The Director of Public Health played a key role and provided guidance, vision, and insight on a weekly basis; was debriefed on process details throughout the pilot; and was able to maintain relationships with all stakeholders. Additionally, the director firmly determined the level of compliance to be 75%. During planning discussions, revenue concerns were addressed and business cases for success were shared. At the conclusion of negotiations, vending operators held firm in their request to receive a subsidy for potential losses. This subsidy requirement illustrates the balance and compromise needed for a collaborative initiative to move forward.

The processes of the vending industry, from supply chain to distribution, worked in our favor at the southern site. These machines easily complied with the 75% goal, and few issues arose during the pilot program in sustaining the 75% goal. Often, at the other sites, the food supply company made unapproved product substitutions, incorrectly stocked the machines according to the healthy and pre-approved planogram, or no longer carried a particular item in the warehouse. Not all parties in distribution channels were aware of, or agreed to, pilot goals. The substitution of incorrect products required additional project management oversight and ultimately accounted for a majority of machine noncompliance.

We did not find substantial reductions in the number of units sold overall from the previous year; sales reductions were a concern of the vendors during the planning process. At the southern site, vending machine profits were 51% higher in January 2012 than profits averaged at that site for January 2011 and January 2010, and profits there were above average levels for 5 of 6 months of the pilot program. However, under the agreement between the Business Enterprise Program and the Division of Public Health, the vending operators were compensated a total of $1,383 during the pilot for overall revenue loss and product spoilage caused by the shorter shelf life of more healthful items.

Additional research is needed to understand customer preferences. Although general customer preference and taste surveys were conducted before the pilot program, additional research could determine whether new customers were enticed to use the vending machines or whether repeat and existing customers were purchasing more healthful items than before. Insight into customer preferences can help in the tailoring of healthful product offerings, thereby theoretically motivating the customer to use the machine, make healthful food purchases, and increase sales revenue for the machine’s owner. Our pilot program engaged customers through new, more healthful product taste testing, online taste preferences surveys, and a series of e-mails featuring a more healthful product and health facts (eg, “Sugary beverages like regular soda, sweetened teas, and energy drinks provide little or no nutritional benefit and lots of empty calories. Replacing one regular soda that contains 10 teaspoons of sugar a day with water can save you 150 calories or 15 pounds per year!”).

To support and sustain a component of healthy behavior change, contract terms must be in place to support healthy vending. Although our pilot program successfully operated outside of the regular contract negotiation process, we recommend that healthful vending specifications be included in a formal contract with a food service provider so that projects can be sustained. Many models exist around the nation, and Nemours Healthy Vending and Concessions Guidelines can also serve as a tool in defining healthful specifications for any food service operation (9,11). In 2012, these guidelines were used to inform the US General Services Administration for revision of food and beverage standards for contracted cafeteria, concession, and vending services on federal property (12).

Our evaluation has several limitations. We did not have data on vending machines in buildings that did not participate in the pilot, so it is possible that changes in profits from the machines in the pilot program could have occurred had the pilot program not been implemented. Furthermore, research suggests that comprehensive workplace health promotion efforts hold the most promise for significant change in healthful eating and physical activity (13); this program included only one component. If additional workplace efforts related to healthful eating had been implemented at these sites, we may have found different outcomes. In addition, changes were made to the prices of unhealthful food and beverage items (eg, receiving a larger size candy bar for a reduced price and reducing the price of sodas) just before the pilot program began; the influence of those changes on net profit is unknown.
